# “You have the right to protect your health”: Perceptions of Secondhand Smoke and Exposure Mitigation Strategies in Low-Income Patients With Heart Disease, San Francisco, 2011–2012

**DOI:** 10.5888/pcd13.150593

**Published:** 2016-08-25

**Authors:** Cati G. Brown-Johnson, Marily Oppezzo, Neal L. Benowitz, Judith J. Prochaska

**Affiliations:** Author Affiliations: Cati G. Brown-Johnson, Stanford Prevention Research Center, and Evaluation Sciences Unit, Department of Medicine, Stanford University, Stanford, California; Marily Oppezzo, Stanford Prevention Research Center, Department of Medicine, Stanford University, Stanford, California; Neal L. Benowitz, Departments of Medicine and Bioengineering and Therapeutic Sciences, Division of Clinical Pharmacology and Experimental Therapeutics, University of California, San Francisco, San Francisco, California.

## Abstract

We examined the understanding of the harms of secondhand smoke (SHS) exposure among low-income, hospitalized adults with cardiovascular disease. Participants were 15 nonsmokers reporting daily SHS exposure and 15 light or nondaily cigarette smokers. We coded responses from audiotaped semistructured interviews for themes. No participant spontaneously identified heart risks related to SHS exposure. Strategies to avoid SHS included verbal requests to not smoke and physically avoiding smoke; both smokers and nonsmokers prioritized politeness over urgency. Most participants thought a blood test quantifying SHS exposure would be clinically useful. Health education, assertiveness communication training, and protective policies (eg, smoke-free multiunit housing) also were supported.

## Objective

Secondhand smoke (SHS) accounts for 41,000 US deaths annually, more than 80% of which result from cardiovascular disease (CVD) (1). Although the prevalence of daily smoking has declined, any level of tobacco smoke exposure brings serious health consequences (1). With CVD, heavy SHS and light or nondaily smoking have negative and comparable levels of harm (2). However, medical systems rarely assess or provide interventions for intermittent tobacco use or SHS exposure (3).

Few studies have examined interventions to reduce SHS exposure in adult CVD patients. One quasi-experimental study in middle-income nonsmokers with CVD reported increased awareness of SHS risk; the study did not report change in behavior or biomarkers of exposure (4). Our randomized study with nondaily smokers found that messages focused on SHS harms to others led to greater cotinine-confirmed abstinence compared with the traditional emphasis of tobacco’s harms to self (5).

Tobacco use and SHS exposure are associated with poverty (6,7). To inform innovations to address tobacco-related health disparities, we interviewed uninsured patients with CVD recruited from a public hospital. The sample consisted of light (<5 cigarettes/d) and nondaily (<7 d/wk) cigarette smokers and nonsmokers exposed to SHS.

## Methods

The study was conducted from April 2011 through May 2012 on the cardiology service at San Francisco General Hospital, a large, urban public hospital that serves an ethnically diverse and low-income population. We recruited 15 nonsmokers who reported daily SHS exposure before hospitalization and 15 light/nondaily cigarette smokers. Institutional review boards approved study procedures, and participants provided informed consent.

Study procedures were performed in-hospital, averaged 1.5 hours, and included a half-hour structured interview on SHS risk perceptions and strategies to reduce SHS exposure. When available, we analyzed blood samples obtained at hospital admission for cotinine.

We transcribed and analyzed the audiotaped interviews using a general inductive approach (8). The first 2 authors (C. G. B., M. O.) independently coded structured queries with good agreement (Cohen’s κ = 0.76). Emergent themes captured strategies to reduce SHS exposure. Two research assistants independently coded the transcripts with moderate to high interrater reliability (Cohen’s κ = 0.66–0.75). The first author resolved coding conflicts.

## Results

Participant demographics did not differ by smoking status. The sample (N = 30) was primarily male (n = 25) and racially/ethnically diverse (African American, n = 13; Asian/Pacific Islander, n = 4; white, n = 7; multiracial/other, n = 6). Before hospitalization, most patients were unhoused (n = 8) or residing in hotels or single-room occupancy units (n = 9). Most nonsmokers were former smokers (n = 12). Most smokers (n = 12) wanted to quit; only 4 were prepared to quit within the month. Blood samples were available for 9 participants; cotinine values averaged 8.66 ng/ml (standard deviation, 11.58 ng/ml; range, 0.33–34.88 ng/ml).

Most understood SHS to be harmful (Table 1); however, without prompting, not one participant identified adverse effects on the heart. After prompting, 19 linked SHS to heart outcomes, although comprehension of SHS effects on the heart was neither uniform nor always accurate. Although 8 participants considered SHS equal to or worse than smoking (27%), 3 said it was less harmful. Many smokers (n = 6) reported concern about the effects of SHS on others. All nonsmoking patients and 13 smokers (87%) thought use of a blood test to quantify recent SHS exposure would increase risk awareness, motivate self-care, and help light/nondaily smokers “cut back.”

**Table 1:** Secondhand Smoke (SHS) Perceptions of Nonsmokers and Light/Nondaily Cigarette Smokers, San Francisco, 2011–2012

Characteristic	Nonsmoker (n = 15)	Light/Nondaily Smoker^a^ (n = 15)
	No. (%)	
**Perceptions of SHS**		
**Spontaneously identified health risks^b^**	13 (93)	12 (80)
General illness	6 (43)	6 (40)
Lung disease (emphysema, asthma, bronchitis)	4 (29)	5 (33)
Cancer	4 (29)	4 (27)
Death	2 (14)	2 (13)
**With prompting, acknowledged SHS heart risks^b^**	9 (64)	10 (67)
**Nonhealth-related perceptions of SHS**		
Nuisance	12 (80)	9 (60)
Bad smell	4 (27)	0

a Light smokers smoked fewer than 5 cigarettes per day and nondaily smokers smoked cigarettes weekly but not every day. Participants gave multiple responses in a semi-structured interview tapping their perceptions of the risks of SHS exposure. Less commonly identified health risks (n<4) were throat problems (n = 2), nausea (n = 2), bad for brain/difficult to think (n = 2), toxic blood levels (n = 1), irritability (n = 1), eye irritation (n = 1), and addiction (n = 1). No participants spontaneously mentioned adverse effects of SHS exposure on the heart.

b Reponses to questions about health risks provided by 14 of 15 nonsmokers.

When asked how to reduce SHS exposure, participants’ suggestions focused on communication, information, physical avoidance, policy strategies, and biomarker feedback (Table 2). Both smokers and nonsmokers emphasized politeness and respect; a minority of nonsmokers suggested more forceful verbal and physical actions. Some smokers reported that a simple “please” prompted them to move elsewhere, extinguish their cigarette, or not smoke the entire day. A “please” coupled with harsh language was still perceived as respectful. Other strategies for avoiding SHS included giving personal health reasons when asking others not to smoke (n = 4) and invoking the presence of children (n = 3). One participant stated that children trumped the need for politeness.

**Table 2:** Reported Strategies to Avoid Secondhand Smoke Exposure Among Nonsmokers and Light/Nondaily Cigarette Smokers, San Francisco, 2011–2012^a^

Strategies^b^	Nonsmokers (n = 15)	Light/Nondaily Smokers (n = 15)	Sample Quotes
	No. (%)		
**Communication approaches^c^**	15 (100)	11 (73)	** —**
Conversational (with friends/family)	12 (80)	9 (60)	Say, “Hey, well you’re married to me, and you’re part of my life and you are killing me [with SHS].” NS4
Polite/sincere	13 (87)	7 (47)	They said, “Please don’t smoke near me” . . . Yeah, politely. “Please put that cigarette out.” LNDS2 I said, “I can’t tell you what to do, but can you move over away from me?” NS8
Demanding/forceful	7 (47)	6 (40)	“You ain’t smoking in here.” LNDS6 “Put that f-ing cigarette out.” NS3
Threats of violence	2 (13)	0	“Threaten them [smokers].” NS7
**Information^d^**	9 (60)	6 (40)	** —**
Invoke smoke-free rule	5 (33)	5 (33)	“Look there’s no smoking in the house” . . . that was understood. It’s an unwritten rule. LNDS5
Invoke children	2 (13)	1 (7)	Try to talk with reason: “You can’t smoke ’cause my son’s got asthma.” NS4 Moms can be direct . . . [they] don’t need to be kind. LNDS8
State personal reasons	2 (13)	2 (13)	I just went straight and said, “Well I’m very sick, please don’t smoke near me.” LNDS7
Educate on SHS risks	3 (20)	1 (7)	I just told him, “You know, even secondhand smoking kills.” LNS13
**Physical strategies^e^**	7 (47)	1 (7)	** —**
Better ventilation or filtration	2 (7)	0	Open a window. NS5 [A personal air filter] . . . handy pack on the shoulder strap to breathe oxygen. NS11
Move away from SHS	6 (40)	0	If I see somebody smoking, I try to walk far away from them. NS4
Avoid smokers	2 (13)	1 (7)	Don’t hang around people that smoke. LNDS14
**Policy^f^**	4 (27)	3 (27)	** —**
Smoke-free multiunit housing	2 (13)	0	If I had the money, then I would build a building where smoking isn’t allowed — period. NS9
Smoke-free signage and designated smoking areas	2 (13)	3 (20)	. . . put signs telling you where to smoke and where not to smoke. LNDS6
**Smoke exposure blood test**	15 (100)	13 (87)	I’ll know how much I’ve been exposed to [with a blood test], so I will try to . . . quit using cigarettes. LNDS2

Abbreviations: — , not applicable; LNDS, light nondaily smoker; NS, nonsmoker; SHS, secondhand smoke.

a Percentages may exceed 100% within an area, because participants could list more than 1 strategy.

b Using a general inductive approach, we determined categories for strategies by describing all strategies, and exploring how those descriptions were similar or different for a final reduction to 4 thematic categories: communication, information strategy, physical strategy, and policy.

c Communication approaches focused on how and in what way a person might communicate with other people to best avoid SHS exposure. All communication approaches were verbal, including threats of violence.

d Information strategies focused on what information was being communicated instead of how communication happened.

e Physical approaches focused on strategies to directly manipulate the physical world, including objects or bodies.

f Policy strategies included reference to rules and policies that might affect more than one person.

Strategies emphasizing participants’ physical agency (ie, moving away from SHS) were less common, suggested by 7 nonsmokers and 1 light smoker. SHS exposure in single-room occupancy hotel-like settings was common, regardless of participants’ personal home smoking rules; smoke-free multiunit housing policies were encouraged (n = 2), and one participant dreamed of building smoke-free apartments.

## Discussion

The study findings indicate SHS knowledge gaps in a low-income sample of CVD patients. Although most participants in our study identified SHS exposure as a nuisance and harmful to health, not one participant spontaneously listed heart disease as an SHS risk. There was greater awareness that SHS causes lung disease and some cancers. Although these are also serious health concerns, SHS effects on the heart are more immediate, acute, and relevant to study participants’ hospitalization on a cardiology service (9).

Both nonsmokers and light/nondaily smokers reported motivation to avoid SHS and use of similar techniques to protect themselves from tobacco smoke. Most thought a blood test quantifying SHS exposure would motivate assertive communications and avoidance behaviors, provide useful data to support protective policies (eg, smoke-free multiunit housing), and raise motivation to quit among light/nondaily smokers. A biomarker test would align with calls to integrate behavioral data for personalized medical care (10), and prompt intervention with light/nondaily smokers, often overlooked in cessation counseling (3). In practice, availability of SHS blood tests is influenced by cost and treatment prioritization.

Participants residing in low-income multiunit housing reported low perceived control over building-level clean-air policies. A 2015 review concluded the evidence is sufficient to support multiunit housing smoke-free policies on a broad scale (11). SHS interventions should advise home smoking bans and refer to local action networks that support clean-air policies.

The study sample was limited in size, geographic region, language (English-speaking), and socioeconomic status. The findings, however, are novel and informative for future treatment efforts. Patients with CVD may lack critical information connecting their light/nondaily smoking and SHS exposure to immediate heart risks. Furthermore, low-income patients may experience SHS exposure as a result of environmental and residential factors. Extended clean-air policies in public (eg, worksites) and private (eg, multiunit housing) environments are needed to protect communities at all income strata. Individualized interventions should address the immediate risk of SHS exposure for low-income patients with CVD.
